# Poly[μ_3_-acetato-di-μ_3_-isonicotinato-μ_2_-isonicotinato-samarium(III)silver(I)]

**DOI:** 10.1107/S1600536809048430

**Published:** 2009-11-21

**Authors:** Li-Cai Zhu

**Affiliations:** a School of Chemistry and Environment, South China Normal University, Guangzhou 510631, People’s Republic of China

## Abstract

In the title homochiral three-dimensional heterometallic complex, [AgSm(C_6_H_4_NO_2_)_3_(C_2_H_3_O_2_)]_*n*_, the eight-coordinate Sm^III^ ion displays a bicapped trigonal-prismatic geometry, being coordinated by two O atoms from one acetate ligand, four O atoms from four bridging isonicotinate ligands and two O atoms from two terminal isonicotinate ligands. The four-coordinate Ag^I^ ion adopts a tetra­hedral geometry, being bonded to two N atoms from two bridging isonicotinate ligands and two O atoms from two acetate ligands. These metal coordination units are connected by bridging isonicotinate and acetate ligands, generating a three-dimensional network.

## Related literature

For the applications of lanthanide–transition metal heterometallic complexes with bridging multifunctional organic ligands in ion exchange, magnetism, bimetallic catalysis and as luminescent probes, see: Cheng *et al.* (2006 ▶); Gu & Xue (2006 ▶); Peng *et al.* (2008 ▶); Zhu *et al.* (2009 ▶).
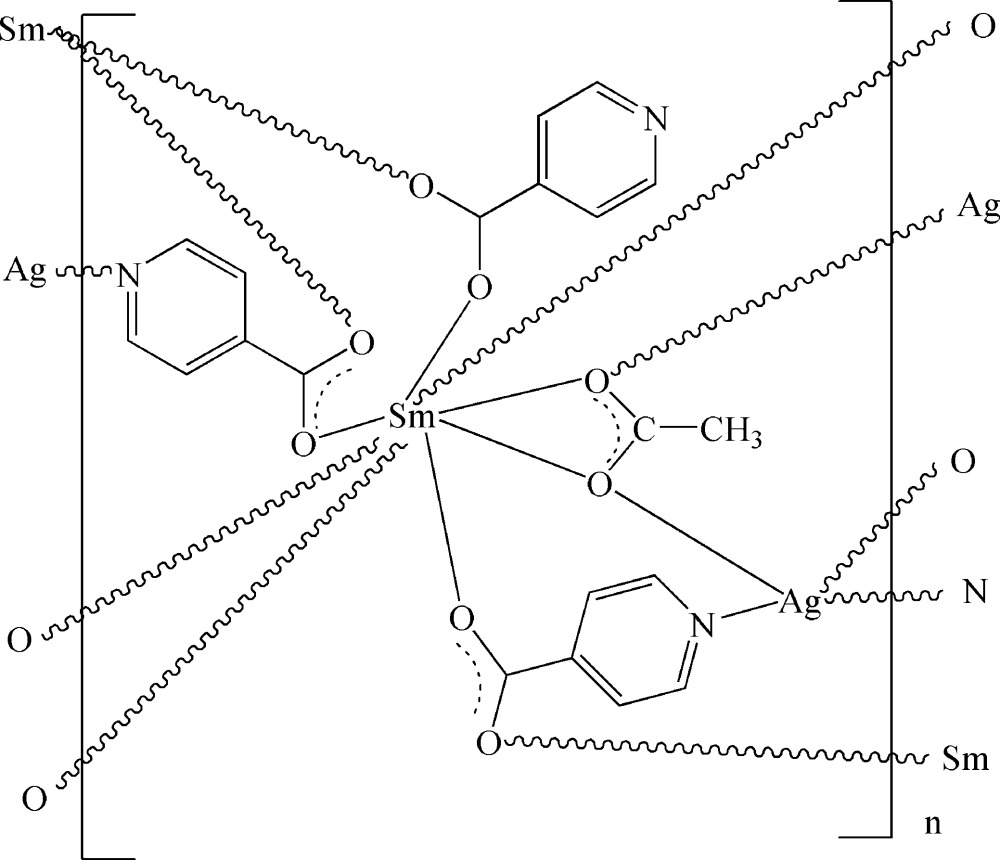


## Experimental

### 

#### Crystal data

[AgSm(C_6_H_4_NO_2_)_3_(C_2_H_3_O_2_)]
*M* *_r_* = 683.58
Hexagonal, 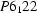

*a* = 11.8184 (5) Å
*c* = 27.340 (2) Å
*V* = 3307.0 (3) Å^3^
*Z* = 6
Mo *K*α radiation
μ = 3.58 mm^−1^
*T* = 296 K
0.23 × 0.20 × 0.19 mm

#### Data collection

Bruker APEXII area-detector diffractometer
Absorption correction: multi-scan (*SADABS*; Sheldrick, 1996 ▶) *T*_min_ = 0.444, *T*_max_ = 0.507
17136 measured reflections
1992 independent reflections
1928 reflections with *I* > 2σ(*I*)
*R*_int_ = 0.046

#### Refinement

*R*[*F* ^2^ > 2σ(*F* ^2^)] = 0.020
*wR*(*F* ^2^) = 0.049
*S* = 1.06
1992 reflections
154 parameters
H-atom parameters constrained
Δρ_max_ = 0.65 e Å^−3^
Δρ_min_ = −0.45 e Å^−3^
Absolute structure: Flack (1983 ▶), 739 Friedel pairs
Flack parameter: 0.006 (15)

### 

Data collection: *APEX2* (Bruker, 2004 ▶); cell refinement: *SAINT* (Bruker, 2004 ▶); data reduction: *SAINT*; program(s) used to solve structure: *SHELXS97* (Sheldrick, 2008 ▶); program(s) used to refine structure: *SHELXL97* (Sheldrick, 2008 ▶); molecular graphics: *XP* in *SHELXTL* (Sheldrick, 2008 ▶); software used to prepare material for publication: *SHELXL97*.

## Supplementary Material

Crystal structure: contains datablocks I, global. DOI: 10.1107/S1600536809048430/pv2235sup1.cif

Structure factors: contains datablocks I. DOI: 10.1107/S1600536809048430/pv2235Isup2.hkl

Additional supplementary materials:  crystallographic information; 3D view; checkCIF report

## Figures and Tables

**Fig. 1.**
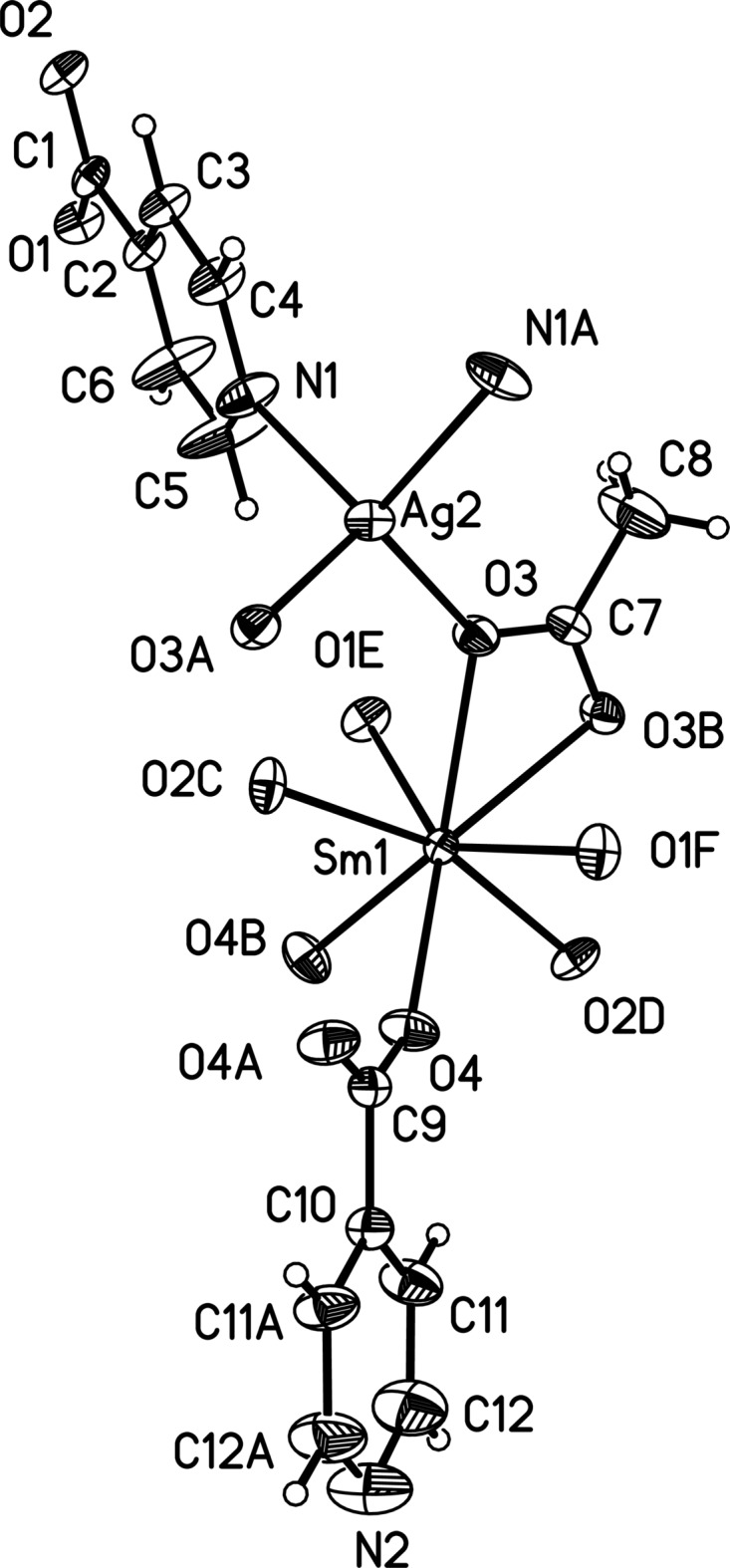
The molecular structure showing the atomic-numbering scheme and displacement ellipsoids drawn at the 30% probability level. Symmetry codes: (A) x, 1 + x-y, 1/6 - z; (B) 1 + x-y, 2 - y, -z; (C) 1 + x-y, 1 - y, -z; (D) 1 + x, 1 + y, z; (E) 1 - y, 1 - x, -1/6 - z; (F) 1 + x-y, 1 + x, 1/6 + z.

**Fig. 2.**
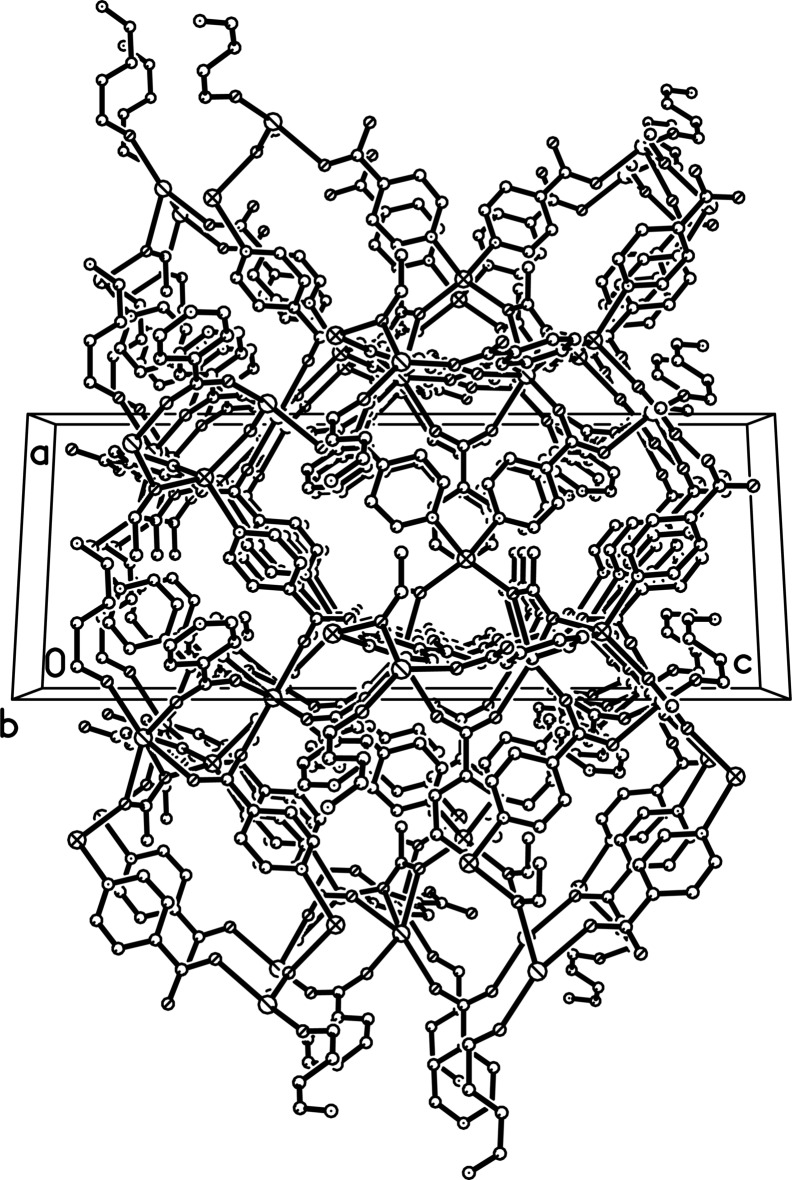
A view of the three-dimensional structure of the title compound. Hydrogen atoms are omitted for clarity.

## supplementary crystallographic information

### Comment

In the past few years, lanthanide-transition metal heterometallic complexs with bridging multifunctionnal organic ligands are of increasing interest, not only because of their impressive topological structures, but also due to their versatile applications in ion exchange, magnetism, bimetallic catalysis and luminescent probe (Cheng *et al.*, 2006; Peng *et al.*, 2008; Zhu *et al.*, 2009). However, because of complicated interactions among the organic moiety and two types of metal centers, the construction of a homochiral Ln—*M* heterometallic coordination framework is one of the most challenging issues in synthetic chemistry and materials science(Gu & Xue, 2006). As an extension of this research, the structure of the title compound, a new homochiral heterometallic coordination polymer, (I), has been determined which is presented in this article.

In the title compound (Fig. 1), there are half of Sm^III^ ion, half of Ag^I^ ion, half of acetate ligand, and one and half crystallogaphically unique isonicotinate ligands in the asymmetrical unit. Isonicotinate ligands have two types of distinctly different coordination modes: one acts as a bridging ligand to coordinate one Ag^I^ ion and two Sm^III^ ions, and the other acts as a terminal ligand to coordinate two Sm^III^ ions. Acetate ligand adopts chelating [Sm^III^] and bridging [Ag^I^] coordination modes. Each Sm^III^ ion is eight-coordinated by two O atoms from one acetate ligand, four O atoms from four bridging isonicotinate ligands, and two O atom from two terminal isonicotinate ligands. The Sm center can be described as having a bicapped trigonal prism coordination geometry. The four-coordinated Ag^I^ ion is bonded to two N atoms from two bridging isonicotinate ligand and two O atoms from two acetate ligands to furnish a tetrahedral geometry, (Table 1). These metal coordination units are connected by bridging isonicotinate and acetate ligands, generating a three-dimensional network (Fig. 2).

### Experimental

A mixture of AgNO_3_(0.057 g, 0.33 mmol), Sm_2_O_3_(0.116 g, 0.33 mmol), isonicotinic acid (0.164 g, 1.33 mmol), acetic acid (0.080 g, 1.33 mmol), and H_2_O(7 ml) was sealed in a 20 ml Teflon-lined reaction vessel at 443 K for 6 days then slowly cooled to room temperature. The product was collected by filtration, washed with water and air-dried. Colorless block crystals suitable for X-ray analysis were obtained.

### Refinement

All H atoms bonded to C atoms were positioned geometrically and refined as riding, with C—H = 0.93 or 0.96 Å and *U*_iso_(H) = 1.2 or 1.5 *U*_eq_(C).

### Crystal data

**Table:**

[AgSm(C_6_H_4_NO_2_)_3_(C_2_H_3_O_2_)]	*D*_x_ = 2.059 Mg m^−^^3^
*M**_r_* = 683.58	Mo *K*α radiation, λ = 0.71073 Å
Hexagonal, *P*6_1_22	Cell parameters from 7353 reflections
Hall symbol: P 61 2 (0 0 -1)	θ = 2.5–27.4°
*a* = 11.8184 (5) Å	µ = 3.58 mm^−^^1^
*c* = 27.340 (2) Å	*T* = 296 K
*V* = 3307.0 (3) Å^3^	Block, colorless
*Z* = 6	0.23 × 0.20 × 0.19 mm
*F*(000) = 1974	

### Data collection

**Table:**

Bruker APEXII area-detector diffractometer	1992 independent reflections
Radiation source: fine-focus sealed tube	1928 reflections with *I* > 2σ(*I*)
graphite	*R*_int_ = 0.046
φ and ω scan	θ_max_ = 25.2°, θ_min_ = 2.0°
Absorption correction: multi-scan (*SADABS*; Sheldrick, 1996)	*h* = −13→14
*T*_min_ = 0.444, *T*_max_ = 0.507	*k* = −11→14
17136 measured reflections	*l* = −32→30

### Refinement

**Table:**

Refinement on *F*^2^	Secondary atom site location: difference Fourier map
Least-squares matrix: full	Hydrogen site location: inferred from neighbouring sites
*R*[*F*^2^ > 2σ(*F*^2^)] = 0.020	H-atom parameters constrained
*wR*(*F*^2^) = 0.049	*w* = 1/[σ^2^(*F*_o_^2^) + (0.0242*P*)^2^ + 2.1731*P*] where *P* = (*F*_o_^2^ + 2*F*_c_^2^)/3
*S* = 1.06	(Δ/σ)_max_ = 0.001
1992 reflections	Δρ_max_ = 0.65 e Å^−^^3^
154 parameters	Δρ_min_ = −0.45 e Å^−^^3^
0 restraints	Absolute structure: Flack (1983), 739 Friedel pairs
Primary atom site location: structure-invariant direct methods	Flack parameter: 0.006 (15)

### Special details

**Table:**

Geometry. All e.s.d.'s (except the e.s.d. in the dihedral angle between two l.s. planes) are estimated using the full covariance matrix. The cell e.s.d.'s are taken into account individually in the estimation of e.s.d.'s in distances, angles and torsion angles; correlations between e.s.d.'s in cell parameters are only used when they are defined by crystal symmetry. An approximate (isotropic) treatment of cell e.s.d.'s is used for estimating e.s.d.'s involving l.s. planes.
Refinement. Refinement of *F*^2^ against ALL reflections. The weighted *R*-factor *wR* and goodness of fit *S* are based on *F*^2^, conventional *R*-factors *R* are based on *F*, with *F* set to zero for negative *F*^2^. The threshold expression of *F*^2^ > σ(*F*^2^) is used only for calculating *R*-factors(gt) *etc*. and is not relevant to the choice of reflections for refinement. *R*-factors based on *F*^2^ are statistically about twice as large as those based on *F*, and *R*- factors based on ALL data will be even larger.

### Fractional atomic coordinates and isotropic or equivalent isotropic displacement parameters (Å^2^)

**Table:**

	*x*	*y*	*z*	*U*_iso_*/*U*_eq_	Occ. (<1)
Sm1	0.86531 (2)	1.0000	0.0000	0.02353 (8)	
Ag2	0.51785 (4)	0.75893 (2)	0.0833	0.03675 (12)	
O1	0.1435 (3)	0.2764 (3)	−0.10025 (9)	0.0409 (7)	
O2	−0.0057 (3)	0.1970 (3)	−0.04112 (10)	0.0401 (7)	
O3	0.6346 (3)	0.9170 (3)	0.02435 (11)	0.0401 (7)	
O4	1.0601 (3)	1.0664 (3)	0.04589 (11)	0.0495 (8)	
C1	0.1062 (4)	0.2765 (4)	−0.05754 (14)	0.0312 (10)	
C2	0.1996 (4)	0.3805 (4)	−0.02321 (13)	0.0328 (9)	
C3	0.1670 (4)	0.3897 (4)	0.02451 (15)	0.0410 (12)	
H4	0.0847	0.3306	0.0366	0.049*	
C4	0.2570 (5)	0.4864 (5)	0.05370 (16)	0.0460 (11)	
H2	0.2328	0.4914	0.0856	0.055*	
C5	0.4073 (6)	0.5645 (7)	−0.0058 (2)	0.101 (3)	
H3	0.4902	0.6253	−0.0169	0.121*	
C6	0.3228 (6)	0.4690 (6)	−0.03802 (19)	0.083 (3)	
H5	0.3500	0.4655	−0.0696	0.100*	
C7	0.6215 (5)	1.0000	0.0000	0.0345 (13)	
C8	0.4962 (7)	1.0000	0.0000	0.089 (3)	
H11A	0.5133	1.0884	−0.0010	0.133*	0.50
H11B	0.4481	0.9581	0.0292	0.133*	0.50
H11C	0.4458	0.9535	−0.0282	0.133*	0.50
C9	1.1136 (5)	1.0568 (3)	0.0833	0.0345 (13)	
C10	1.2622 (6)	1.1311 (3)	0.0833	0.0375 (13)	
C11	1.3318 (5)	1.2039 (6)	0.04352 (19)	0.0576 (15)	
H13	1.2889	1.2083	0.0157	0.069*	
C12	1.4659 (6)	1.2700 (8)	0.0456 (3)	0.082 (2)	
H14	1.5112	1.3200	0.0186	0.099*	
N1	0.3766 (4)	0.5734 (4)	0.03960 (13)	0.0508 (11)	
N2	1.5358 (7)	1.2679 (4)	0.0833	0.093 (3)	

### Atomic displacement parameters (Å^2^)

**Table:**

	*U*^11^	*U*^22^	*U*^33^	*U*^12^	*U*^13^	*U*^23^
Sm1	0.02697 (12)	0.02447 (15)	0.01831 (13)	0.01224 (7)	0.00126 (6)	0.00253 (11)
Ag2	0.0309 (2)	0.0440 (2)	0.0310 (2)	0.01544 (12)	0.000	−0.00131 (18)
O1	0.0433 (18)	0.0454 (18)	0.0227 (14)	0.0137 (15)	−0.0043 (13)	−0.0073 (12)
O2	0.0395 (17)	0.0320 (16)	0.0298 (15)	0.0036 (14)	−0.0050 (13)	0.0045 (12)
O3	0.0345 (16)	0.0457 (18)	0.0413 (17)	0.0210 (14)	0.0125 (13)	0.0158 (14)
O4	0.0354 (17)	0.070 (2)	0.0403 (17)	0.0244 (15)	−0.0079 (14)	0.0031 (15)
C1	0.038 (2)	0.024 (2)	0.024 (2)	0.0092 (18)	−0.0050 (17)	0.0024 (16)
C2	0.038 (2)	0.028 (2)	0.023 (2)	0.009 (2)	−0.0038 (18)	0.0002 (17)
C3	0.040 (3)	0.033 (2)	0.031 (2)	0.004 (2)	0.0042 (18)	−0.0024 (19)
C4	0.048 (3)	0.042 (3)	0.028 (2)	0.008 (2)	0.002 (2)	−0.008 (2)
C5	0.061 (4)	0.095 (5)	0.045 (3)	−0.037 (3)	0.022 (3)	−0.033 (3)
C6	0.066 (4)	0.075 (4)	0.031 (3)	−0.023 (3)	0.017 (3)	−0.020 (3)
C7	0.031 (2)	0.046 (3)	0.031 (3)	0.0231 (17)	−0.0010 (15)	−0.002 (3)
C8	0.062 (3)	0.129 (9)	0.098 (7)	0.064 (4)	0.021 (3)	0.042 (7)
C9	0.031 (3)	0.034 (2)	0.037 (3)	0.0156 (16)	0.000	0.000 (2)
C10	0.035 (3)	0.042 (3)	0.034 (3)	0.0174 (16)	0.000	0.004 (3)
C11	0.041 (3)	0.080 (4)	0.051 (3)	0.029 (3)	0.011 (2)	0.025 (3)
C12	0.055 (4)	0.102 (6)	0.075 (4)	0.028 (4)	0.026 (3)	0.027 (4)
N1	0.042 (2)	0.046 (2)	0.033 (2)	−0.0024 (19)	−0.0019 (18)	−0.0115 (17)
N2	0.049 (4)	0.120 (6)	0.086 (6)	0.025 (2)	0.000	0.009 (5)

### Geometric parameters (Å, °)

**Table:**

Sm1—O2^i^	2.336 (3)	C3—H4	0.9300
Sm1—O2^ii^	2.336 (3)	C4—N1	1.323 (6)
Sm1—O4	2.384 (3)	C4—H2	0.9300
Sm1—O4^iii^	2.384 (3)	C5—N1	1.311 (6)
Sm1—O1^iv^	2.478 (3)	C5—C6	1.386 (7)
Sm1—O1^v^	2.478 (3)	C5—H3	0.9300
Sm1—O3	2.483 (3)	C6—H5	0.9300
Sm1—O3^iii^	2.483 (3)	C7—O3^iii^	1.257 (4)
Ag2—N1	2.316 (4)	C7—C8	1.482 (9)
Ag2—N1^vi^	2.316 (4)	C8—H11A	0.9600
Ag2—O3	2.328 (3)	C8—H11B	0.9600
Ag2—O3^vi^	2.328 (3)	C8—H11C	0.9600
O1—C1	1.248 (5)	C9—O4^vi^	1.238 (4)
O1—Sm1^vii^	2.478 (3)	C9—C10	1.521 (8)
O2—C1	1.261 (5)	C10—C11	1.376 (6)
O2—Sm1^viii^	2.336 (3)	C10—C11^vi^	1.376 (6)
O3—C7	1.257 (4)	C11—C12	1.374 (8)
O4—C9	1.238 (4)	C11—H13	0.9300
C1—C2	1.501 (5)	C12—N2	1.329 (8)
C2—C6	1.362 (7)	C12—H14	0.9300
C2—C3	1.380 (6)	N2—C12^vi^	1.329 (8)
C3—C4	1.363 (6)		
			
O2^i^—Sm1—O2^ii^	162.24 (16)	C9—O4—Sm1	149.2 (3)
O2^i^—Sm1—O4	83.32 (11)	O1—C1—O2	124.9 (4)
O2^ii^—Sm1—O4	82.47 (11)	O1—C1—C2	118.0 (4)
O2^i^—Sm1—O4^iii^	82.47 (11)	O2—C1—C2	117.1 (3)
O2^ii^—Sm1—O4^iii^	83.32 (11)	C6—C2—C3	117.0 (4)
O4—Sm1—O4^iii^	73.52 (16)	C6—C2—C1	120.6 (4)
O2^i^—Sm1—O1^iv^	102.15 (10)	C3—C2—C1	122.4 (4)
O2^ii^—Sm1—O1^iv^	83.83 (10)	C4—C3—C2	119.2 (4)
O4—Sm1—O1^iv^	145.28 (11)	C4—C3—H4	120.4
O4^iii^—Sm1—O1^iv^	73.30 (11)	C2—C3—H4	120.4
O2^i^—Sm1—O1^v^	83.83 (10)	N1—C4—C3	124.3 (4)
O2^ii^—Sm1—O1^v^	102.15 (10)	N1—C4—H2	117.9
O4—Sm1—O1^v^	73.30 (11)	C3—C4—H2	117.9
O4^iii^—Sm1—O1^v^	145.28 (11)	N1—C5—C6	123.5 (5)
O1^iv^—Sm1—O1^v^	141.02 (16)	N1—C5—H3	118.3
O2^i^—Sm1—O3	124.27 (10)	C6—C5—H3	118.3
O2^ii^—Sm1—O3	73.44 (10)	C2—C6—C5	119.6 (5)
O4—Sm1—O3	132.68 (10)	C2—C6—H5	120.2
O4^iii^—Sm1—O3	139.93 (12)	C5—C6—H5	120.2
O1^iv^—Sm1—O3	72.14 (10)	O3^iii^—C7—O3	118.3 (5)
O1^v^—Sm1—O3	72.89 (11)	O3^iii^—C7—C8	120.9 (3)
O2^i^—Sm1—O3^iii^	73.44 (10)	O3—C7—C8	120.9 (3)
O2^ii^—Sm1—O3^iii^	124.27 (10)	O3^iii^—C7—Sm1	59.1 (3)
O4—Sm1—O3^iii^	139.93 (12)	O3—C7—Sm1	59.1 (3)
O4^iii^—Sm1—O3^iii^	132.68 (10)	C8—C7—Sm1	180.000 (1)
O1^iv^—Sm1—O3^iii^	72.89 (11)	C7—C8—H11A	109.5
O1^v^—Sm1—O3^iii^	72.14 (10)	C7—C8—H11B	109.5
O3—Sm1—O3^iii^	51.51 (13)	H11A—C8—H11B	109.5
O2^i^—Sm1—C7	98.88 (8)	C7—C8—H11C	109.5
O2^ii^—Sm1—C7	98.88 (8)	H11A—C8—H11C	109.5
O4—Sm1—C7	143.24 (8)	H11B—C8—H11C	109.5
O4^iii^—Sm1—C7	143.24 (8)	O4—C9—O4^vi^	127.5 (6)
O1^iv^—Sm1—C7	70.51 (8)	O4—C9—C10	116.3 (3)
O1^v^—Sm1—C7	70.51 (8)	O4^vi^—C9—C10	116.3 (3)
O3—Sm1—C7	25.76 (7)	C11—C10—C11^vi^	117.6 (6)
O3^iii^—Sm1—C7	25.76 (7)	C11—C10—C9	121.2 (3)
N1—Ag2—N1^vi^	102.7 (2)	C11^vi^—C10—C9	121.2 (3)
N1—Ag2—O3	105.05 (13)	C10—C11—C12	118.8 (5)
N1^vi^—Ag2—O3	112.42 (14)	C10—C11—H13	120.6
N1—Ag2—O3^vi^	112.42 (14)	C12—C11—H13	120.6
N1^vi^—Ag2—O3^vi^	105.05 (13)	N2—C12—C11	125.0 (6)
O3—Ag2—O3^vi^	118.21 (15)	N2—C12—H14	117.5
C1—O1—Sm1^vii^	124.3 (3)	C11—C12—H14	117.5
C1—O2—Sm1^viii^	146.4 (3)	C5—N1—C4	116.4 (4)
C7—O3—Ag2	137.6 (3)	C5—N1—Ag2	117.8 (3)
C7—O3—Sm1	95.1 (3)	C4—N1—Ag2	124.7 (3)
Ag2—O3—Sm1	126.58 (12)	C12—N2—C12^vi^	114.9 (7)

Symmetry codes: (i) *x*+1, *y*+1, *z*; (ii) *x*−*y*+1, −*y*+1, −*z*; (iii) *x*−*y*+1, −*y*+2, −*z*; (iv) −*y*+1, −*x*+1, −*z*−1/6; (v) *x*−*y*+1, *x*+1, *z*+1/6; (vi) *x*, *x*−*y*+1, −*z*+1/6; (vii) *y*−1, −*x*+*y*, *z*−1/6; (viii) *x*−1, *y*−1, *z*.
